# Hygroscopic bioactive light-cured composite promoting dentine bridge formation

**DOI:** 10.1093/rb/rbae114

**Published:** 2024-09-26

**Authors:** Yunzi Long, Guibin Huang, Siyi Liu, Liju Xu, Ailing Li, Dong Qiu, Yanmei Dong

**Affiliations:** Department of General Dentistry II, Peking University School and Hospital of Stomatology, Beijing 100081, PR China; Department of Cariology and Endodontology, Peking University School and Hospital of Stomatology, Beijing 100081, PR China; Department of Cariology and Endodontology, Peking University School and Hospital of Stomatology, Beijing 100081, PR China; Beijing National Laboratory for Molecular Sciences, CAS Research/Education Center for Excellence in Molecular Sciences, Institute of Chemistry, Chinese Academy of Sciences, Beijing 100190, PR China; Beijing National Laboratory for Molecular Sciences, CAS Research/Education Center for Excellence in Molecular Sciences, Institute of Chemistry, Chinese Academy of Sciences, Beijing 100190, PR China; Beijing National Laboratory for Molecular Sciences, CAS Research/Education Center for Excellence in Molecular Sciences, Institute of Chemistry, Chinese Academy of Sciences, Beijing 100190, PR China; School of Chemical Sciences, University of Chinese Academy of Sciences, Beijing 10019, PR China; Department of Cariology and Endodontology, Peking University School and Hospital of Stomatology, Beijing 100081, PR China

**Keywords:** bioactive glass, TheraCal LC, direct pulp capping, light-cured, hygroscopic

## Abstract

A light-cured bioactive composite, TheraCal LC, is easy to handle and fast-setting. But poor water absorption restricted its bioactivity when applied in direct pulp capping (DPC). Enhancing the water absorption of resin-based bioactive materials may be key to optimizing biomineralization procedure of light-cured bioactive materials. We constructed a hygroscopic, light-cured bioactive composite made up of bioactive glass (BG), poly (ethylene glycol) (PEG) and resin in this study. BG was encapsulated into a porogen (i.e. PEG) and mixed into resin matrix. Inductively coupled plasma showed that light-cured BG (LC-BG) exhibited faster ion release and more ion exchange than TheraCal LC did. The formation of macropores and hydroxyapatite crystal coatings on the BG microparticles was observed using scanning electron microscopy. The shear bond strength between the resin and LC-BG group did not significantly differ from the TheraCal LC group. CCK-8 assay showed that the LC-BG extract was nontoxic. Real-time polymerase chain reaction revealed that LC-BG upregulated odontogenic gene expression in human dental pulp cells. DPC assay proved that the LC-BG group exhibited no significant difference in dentin tubule formation (*P* = 0.659) or odontoblast-like cell layer formation (*P* = 0.155) from the TheraCal LC group, but exhibited significantly better integrity of the calcified bridge than the TheraCal LC group (*P *=* *0.039); more DSPP-positive and DMP-1-positive cells were detected in the LC-BG group than in the TheraCal LC group. Although no significant difference in pulpal inflammatory cell infiltration was observed between the LC-BG group and the TheraCal LC group (*P* = 0.476), fewer interleukin 1β-positive and tumor necrosis factor α-positive cells were detected in the LC-BG group than in the TheraCal LC group. In conclusion, the newly developed hygroscopic LC-BG composite showed better bioactivity and odontogenic differentiation than the TheraCal LC did *in vitro* and induced better integrity of the calcified bridge than the TheraCal LC did *in vivo*.

## Graphical abstract

**Graphical Abstract:**
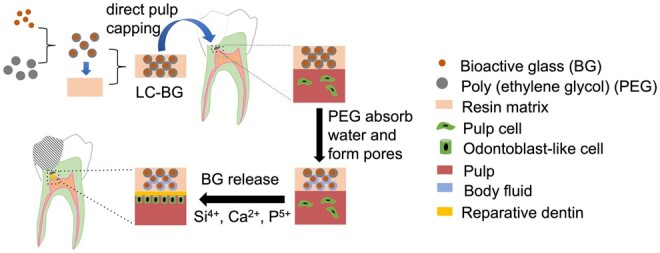

## Introduction

The goal of vital pulp therapy (VPT) is to keep vitality and function of the pulp after injury [1]. Pulp capping materials can influence the prognosis of VPT. Owing to their better bioactivity, calcium silicate cements have a higher success rate when applied to direct pulp capping (DPC) than calcium hydroxide [2–5]. However, calcium silicate cements present several limitations, such as long setting times [6] and poor immediate bond strength to restorations [7].

TheraCal LC (Bisco, USA), a typical light-cured bioactive composite, is made up mainly of Portland cement and resins; its curing time has been shortened to 20s [8], and it is easy to handle because it can be injected directly by a syringe. Some studies have shown that TheraCal LC can improve the proliferation, odontogenic differentiation and mineralization of human dental pulp cells (hDPCs) [9, 10]. Moreover, due to the resin component, TheraCal LC exhibited better bond strength with resin restorations than mineral trioxide aggregate (MTA) [7]. When applied to DPC on human permanent teeth with deep caries, the success rate of TheraCal LC was found to be comparable to that of Dycal (Dentsply, USA) [11, 12]. However, some studies did not support the use of TheraCal LC for DPC because of its poor water absorption ability and impeding the biomineralization procedure [13–18]. Kayad *et al*. [19] reported that TheraCal LC induced slower and thinner dentine bridge formation than Biodentine did after DPC in the incisor of rabbits.

Therefore, enhancing the water absorption of resin-based bioactive materials may be the key point in optimizing the biomineralization procedure. For this consideration, we intended to construct a hygroscopic, light-cured bioactive composite that can absorb water inside the resin and promote biomineralization. A novel BG with high phosphorus content was chosen as the bioactive component because it exhibited fast hydroxyapatite formation ability and excellent bioactivity *in vitro* [20]. In our previous work, the high phosphorus BG has been demonstrated to induce odontogenic differentiation in hDPCs, inhibit the inflammatory response and promote dentin-like tissue formation *in vivo* [21, 22]. Owing to its good hygroscopicity and chemical inertness, poly(ethylene glycol) (PEG) was selected as the water absorbent and porogen to wrap bioactive glass (BG). When in contact with body fluid, PEG can absorb water and dissolve to form pores and tunnels inside the hydrophobic material, providing a moist environment around the BG [23, 24]. We expected that the PEG would absorb body fluid and form pores when contacting pup tissue, creating a moist environment and facilitating BG ion exchange. This study aims to investigate the biological activity of LC-BG *in vitro* and its effect on DPC in rats *in vivo*. The null hypothesis was that there was no difference between LC-BG and TheraCal LC when they were used to DPC.

## Materials and methods

### Materials and reagents

Ca (NO_3_)_2_·4H_2_O and tetraethylorthosilicate were from Sinopharm (China). Glycidyl bisphenol A methacrylate (Bis-GMA), triethylene glycol dimethacrylate (TEGDMA), dimethylaminoethyl methacrylate (DMAEMA) and camphorquinone (CQ) were from Sigma Aldrich (USA). Dulbecco’s modified Eagle’s medium (DMEM), penicillin–streptomycin, L-glutamine and fetal bovine serum were purchased from Gibco (USA). Cell Counting Kit-8 (CCK-8) and the RNA Isolation Kit (R0027, Beyotime, China) were from Beyotime (China).

### Preparation of LC-BG

High phosphorous-containing BG (Wooquick Technology Co., China) is a sol-gel BG (35.0% CaO, 10.8% P_2_O_5_, 54.2% SiO_2_ mol%) and was prepared according to previously described methods [19]. Briefly, phytic acid was first mixed with water and absolute ethanol. Ca (NO_3_)_2_·4H_2_O and tetraethylorthosilicate and were then mixed into the hybrid and sealed to form homogeneous sols. The resulting gels were aged for 24 h at room temperature and dried for 1 week at 60°C and for 2 weeks at 120°C. The dried gels were calcined 1 h under 400°C to fabricate sol-gel BG.

The BG was then encapsulated with PEG. A total of 20.00 g of PEG-5000 powder and 10.00 g of BG particles (size <30 μm) were placed together and heated to 90°C to melt the PEG. After stirring for 2 h, the mixture was cooled and turned back into solid BG/PEG. BG/PEG microparticles (<75 μm) were ground and sieved through 200 mesh sieves. The result of the thermogravimetric analysis of BG/PEG is shown in Supplementary Figure S1.

The resin matrix was prepared as follows: 14.85 g of Bis-GMA was heated to 60°C for 1 h and mixed with 14.85 g of TEGDMA under stirring for 1 h. Then, 0.24 g of DMAEMA and 0.06 g of CQ were mixed into the hybrid under stirring for 24 h in the dark. Then, 30.00 g BG/PEG was added to 30.00 g resin matrix and mixed with an automatic ointment agitator (ARE-310, Thinky, Japan). The final composite is LC-BG.

### Ion concentration and bioactivity *in vitro*

Disk-shaped samples (diameter: 10 mm; height: 1 mm) of LC-BG and TheraCal LC (*n* = 3) were prepared in polytetrafluoroethylene molds. The samples were pressed together by placing the mold between two celluloid strips with two glass microslides on each side. The materials were light-cured with 12 000 W/m^2^ intensity by a LED light-curing unit (Bluephase N, Ivocla) for 30 s each side at a distance of 1 mm. Then, the disks were soaked in 20 ml of simulated body fluid (SBF) (37°C) for 1, 3 and 7 days, and the SBF was not refreshed. The Ca, P and Si concentrations in the LC-BG were detected by inductively coupled plasma optical emission spectroscopy analysis (ICAP 7400; Thermo, USA).

Disk-shaped samples of LC-BG and TheraCal LC (*n* = 3) were prepared as described above. The disks were subsequently soaked in 20 ml of SBF for 1, 3 and 7 days at 37°C, and the SBF was refreshed every 2 days. The solids were longitudinally sectioned and dried at 60°C in a vacuum drying oven for 1 day and then observed by scanning electron microscopy (SEM, SU8010, JEOL, Japan). The crystal structure of minerals deposited on the surface of LC-BG and TheraCal LC was detected by X-ray diffraction (XRD; Rigaku, Japan).

### Degree of conversion

Two pulp capping materials were placed on the surface of the test cell of attenuated total internal reflection Fourier transform infrared spectroscopy (ATR-FTIR; Nexus, America) and then cured for 20 s. ATR-FTIR measurements were carried out before and after light curing. The absorption peaks at 1608 cm^−1^ (corresponding to aromatic C–C reference peak) and 1638 cm^−1^ (corresponding to aliphatic C=C reference peak) were recorded. Three data trials were conducted for each group (1 mm disk). The DC was calculated as follows:

DC=(1-Ab1Ab2Ab3Ab4) * 100%,

where *Ab*1 is the absorbance after light curing at 1638 cm^−1^, *Ab*2 is the absorbance after light curing at 1608 cm^−1^, *Ab*3 is the absorbance before light curing at 1638 cm^−1^ and *Ab*4 is the absorbance before light curing at 1608 cm^−1^.

### Shear bond strength test between pulp capping materials and resin

Twenty cylindrical acrylic molds were prepared with a central hole (internal diameter: 4 mm, height: 2 mm). TheraCal LC and LC-BG were injected into the hole and cured for 1 min. The surfaces of the two materials were polished with 600-mesh abrasive paper. SE Bond (Clearfil™, Japan) was applied to the samples with the following steps: first, apply PRIMER for 20 s and then blow with air flow mildly to remove water completely for 5 s; second, apply BOND and blow gently for 5 s, and then cure for 10 s. A cylindrical-shaped polytetrafluoroethylene mold (internal diameter: 3 mm, height: 3 mm) was placed on the adhesive area. Beautifil Flow Plus F20 (Shofu, Japan) was injected into the mold and bonded to the test materials. The shear bond strength (SBS) was tested by a microshear tester. Shear force was exerted at the material/composite interface until bond failure occurred (speed: 1 mm/min) and the values at failure were recorded. The SBS was calculated as follows:

$$\text{SBS}=F/\pi r^{2},$$

where *F* is the peak fracture load and *r* is the radius of adhesive zone (1.5 mm).

### *In vitro* cytotoxicity assay

The cell experiments were approved by the Peking University School of Stomatology Biomedical Institutional Review Board, Beijing, China (PKUSSIRB-202053006). The test was carried out according to ISO 10993-5: 2009 [25]. Samples (diameter: 10 mm, height: 1 mm) of LC-BG and TheraCal LC were prepared in plastic molds and light-cured for 1 min. All the samples were disinfected for 30 min on each side by ultraviolet light. Samples were then extracted in DMEM at 0.1 g/ml for 3 days at 37°C to obtain the extracts. The material extracts were sterilized by filter with pore size of 0.22 μm. Then 100 U/ml penicillin–streptomycin, 2 mmol/l L-glutamine and 10% fetal bovine serum were added into the extracts for cell culture.

hDPCs were cultured into a 96-well plate (density: 1 × 10^4^ cells/well) overnight. 200 μl dilutions of LC-BG or TheraCal LC extract (undiluted, 2×, 4×, 8×, 16×) were then used to replace the medium, and DMEM was used as a control. The CCK-8 assay was applied at the time point of day 1 to detected cell viability. The value of optical density at 450 nm (630 nm as the reference wavelength) was measured with an Elisa instrument (ELx808; BioTeK, VT).

### Real-time polymerase chain reaction for odontogenic-related gene expression

The hDPCs were cultured into six-well plates (1 × 10^5^ cells/well) and replaced with LC-BG extract, TheraCal LC extract, or DMEM medium when the cells reached 80–90% confluence. At 4 and 7 days, the RNA Isolation Kit was used to isolate the total RNA of hDPCs, and then reverse-transcription and real-time RT-polymerase chain reaction (PCR) were performed. Table 1 shows the primer sequence for target gene (dentin sialophosphoprotein (*DSPP*), dentin matrix protein 1 (*DMP1*) and glyceraldehyde 3-phosphate dehydrogenase (*GAPDH*)). The 2^−ΔΔCt^ method was used to analyze the relative gene expression, and the endogenous reference was GAPDH gene.

**Table 1.** The primers sequences for real-time RT-PCR

Gene	Sequences (F)	Sequences (R)
*DMP1*	CTCCGAGTTGGACGATGAGG	TCATGCCTGCACTGTTCATTC
*DSPP*	ATATTGAGGGCTGGAATGGGGA	TTTGTGGCTCCAGCATTGTCA
*GAPDH*	GAAGGTGAAGGTCGGAGTC	GAGATGGTGATGGGATTTC

### DPC assay

This study was authorized by the Ethics Commission of Peking University Health Science Center, Beijing, China (LA2019358). Laboratory animal technologists were in charge of taking care of the animals, and all the surgery was performed under specific pathogen free (SPF) conditions. The sample size was calculated by *F* tests (analysis of variance (ANOVA): fixed effects, omnibus, one-way) using GPower software version 3.1.9.6. Parameters were set as follows: α-error = 0.05, and power (1-β error) = 0.8, average effect size = 0.73. The results revealed a minimum of nine samples per group.

In the study, 24 upper first molars from 12 male Wistar rats were used (weight: 180–200 g). The surgery was conducted under an OMS2350 dental microscope (Zumax, Suzhou, China) at magnifications of 10.4–17.0 times. The rats were anesthetized with 2% pentobarbital through intraperitoneal injection. The teeth were disinfected with 75% ethanol wipes. A sterilized CAP 3 ultrasonic tip (Satelec, Merignac, France) was used to prepare cavities on the upper first molar’s mesial surface. Mechanical pulp exposures were made by sterile stainless steel files (#15) and irrigated with sterile saline, then pressed on the exposure by sterile paper points for a few seconds to control for bleeding. The pulp exposures were gently capped with LC-BG or TheraCal LC randomly and subsequently restored with Fuji IX (GC, Japan). To minimize occlusal forces, occlusal adjustment was applied to the cusp tip of the opposing teeth, and the rats were fed powdered food.

After 4 weeks, the animals were sacrificed by an excessive anesthesia. The maxillary sections with the first molar were dissected and fixed for 24 h in 4% paraformaldehyde. Then the specimens were decalcified for 4 weeks in 10% EDTA solution and embedded in paraffin. The samples were then serially sectioned at 5 μm thickness in the mesiodistal direction and stained with hematoxylin and eosin. Two inflammatory markers (tumor necrosis factor α (TNF-α) and interleukin 1β (IL-1β)) and two odontogenic markers (DMP1 and DSPP) were conducted for immunohistochemical staining.

Two examiners independently assessed the histological sections according to the criteria presented in Table 2 [26, 27]. Formal assessments were not initiated until the two examiners reached 95% consistency. To avoid possible bias, the specimens were coded. When the two observers had different opinions, they carried out a discussion to reach a consensus.

**Table 2.** Grading of the histologic sections of the pulps tissue after direct pulp capping

	Experimental groups		
Scores	TheraCal LC (*n* = 9)	LC-BG (*n* = 10)	Mann–Whitney test (*P* value)
Calcified bridge formation			
0 = No presence of bridge formation	0	0	0.039^a^
1 = Bridge formation <25%	0	0	
2 = Bridge formation 25–50%	0	0	
3 = Bridge formation 50–75%	1	0	
4 = Bridge formation 75–100%	5	2	
5 = Bridge formation 100%	3	8	
Quality of dentine formation in the bridge			
0 = No tubules present	2	2	0.659
1 = Irregular pattern of tubules	2	4	
2 = Regular pattern of tubules	5	4	
Presence of dentine chips			
0 = No chips	2	3	0.754
1 = Chips	5	5	
2 = Double calcified bridges	2	2	
3 = Pulp stones	0	0	
Pulpal inflammation			
0 = No inflammation	4	5	0.476
1 = Minimal inflammation	2	3	
2 = Moderate inflammation	2	2	
3 = Severe inflammation	1	0	
4 = Abscess formation	0	0	
5 = Tissue necrosis	0	0	
Odontoblast-like cell layer formation			
0 = No odontoblast-like cell layer formation	4	3	0.155
1 = Initial odontoblast-like cell layer extending to <50% of the exposure site	2	2	
2 = Incomplete odontoblast-like cell layer extending to >50% of the exposure site	2	1	
3 = Complete odontoblast-like cell layer formation closing the exposure site completely	1	4	

a Significant difference (*P* < 0.05) between the two groups.

### Statistical analysis

SPSS 26.0 was used for statistical analysis. Independent-samples *t* test was used for statistical analysis of ion release, DC, SBS. One-way ANOVA followed by the multiple-comparison Tukey test was used for CCK-8 assay and RT-PCR. Mann–Whitney *U* test was used for analysis of histological grading of DPC assay. *P *<* *0.05 was considered significant.

## Results

### Ion release

**Figure 1.**
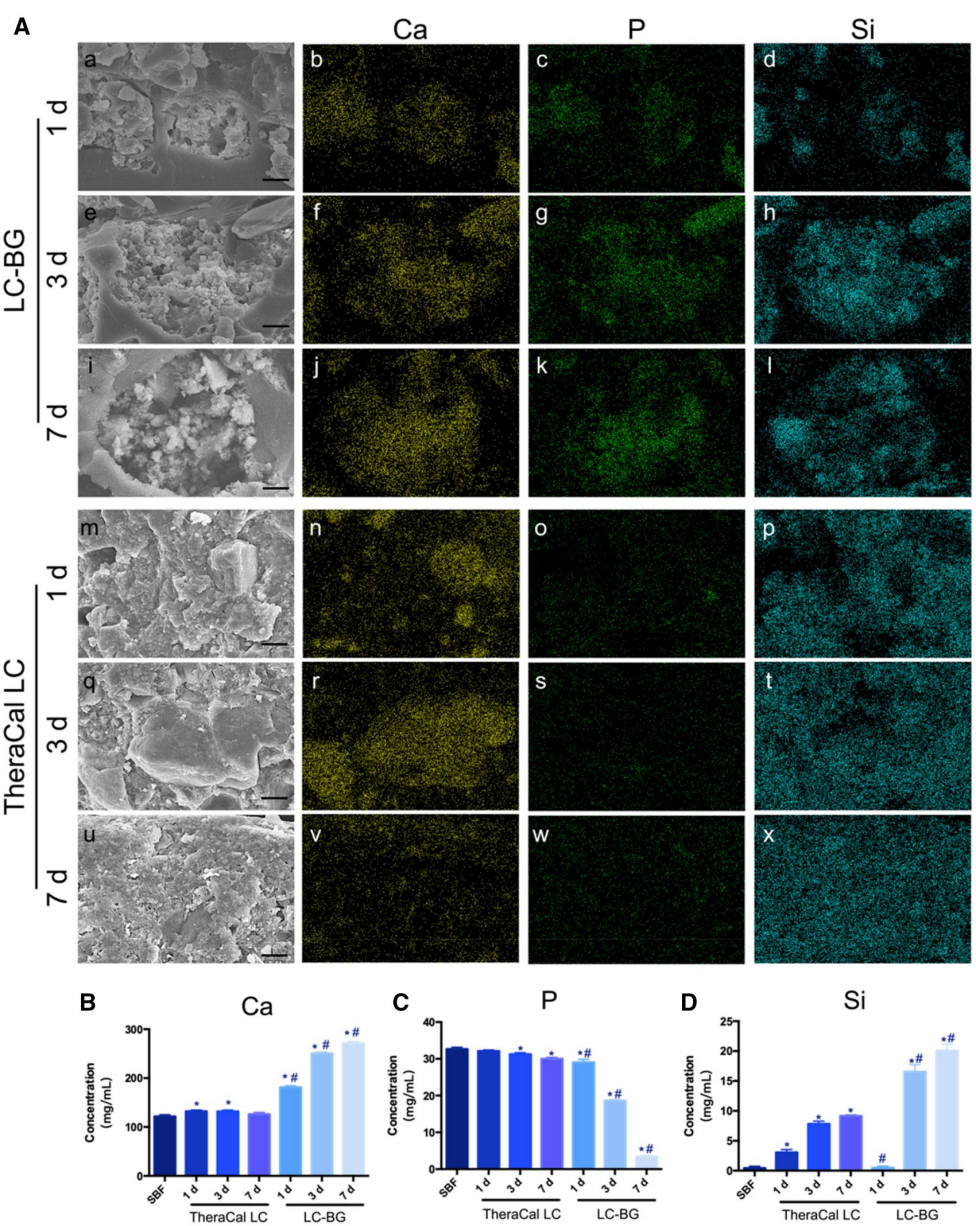
(**A**) SEM photographs (a, e, i, m, q and u) and mapping images of Ca (b, f, j, n, r and v), P (c, g, k, o, s and w) and Si (d, h, l, p, t and x) obtained by EDS (bar: 10 μm). (**B**–**D**) Ion release of Ca, P and Si from the LC-BG and TheraCal LC. *Significant difference compared with the SBF group (*P* < 0.05). ^#^Significant difference compared with the TheraCal LC group at the same concentration (*P* < 0.05).

The results of ion release (Figure 1B and D) showed that the ion concentrations of Ca and Si in the LC-BG extracts significantly increased over time and that the ion concentrations of P (Figure 1C) decreased over time. As to the TheraCal LC group, the ion concentrations of Ca increased on Day 1 but then decreased on Days 3 and 7. The ion concentrations of P decreased over time. Moreover, more Si ions were released on Day 7 in the LC-BG group. No phosphate was detected in the longitudinal section of the TheraCal LC by EDS (Figure 1A, o, s, w), indicating that no hydroxyapatite was generated inside the TheraCal LC. However, calcium and phosphate were detected in the BG particles on Day 7 (Figure 1A, j, k).

### *In vitro* bioactivity

**Figure 2.**
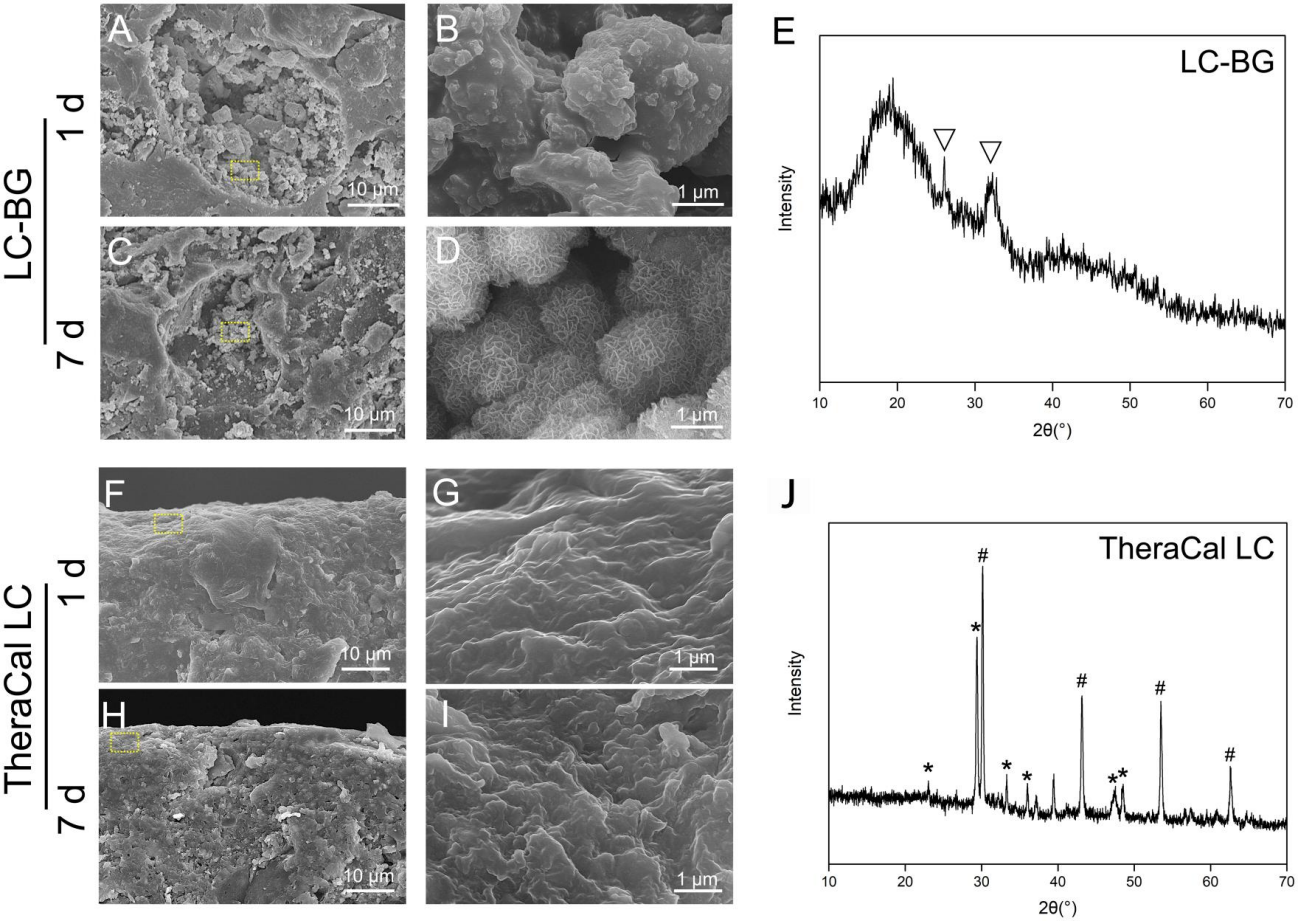
Characterization of LC-BG and TheraCal LC after soaking in SBF. SEM images of longitudinal sections of the LC-BG (**A**–**D**) and TheraCal LC (**F**–**I**). B, D, G and I (bar: 10 μ m) present high-magnification view of rectangular areas in A, C, F, and H (bar: 1 μm). (A–C) Formation of pores inside the LC-BG. (B) No crystals on BG particles. (D) Flake-like HA crystal coating on the BG microparticles. (E) XRD pattern of LC-BG on day 7. ▽: diffraction peaks of hydroxyapatite. (F–I) No pores or obvious hydroxyapatite crystals were observed inside the TheraCal LC. (**J**) XRD pattern of TheraCal LC on day 7. *Diffraction peaks of calcium carbonate; #: diffraction peaks of strontium barium zirconate.

After immersing in SBF for 1 day, the PEG microparticles were removed, and interconnecting pores were observed, but no crystals were found (Figure 2A and B). At 7 days, flake-like hydroxyapatite crystal coatings on the BG microparticles were observed (Figure 2C and D). These findings were in accordance with the XRD results, in which diffraction peaks (26° and 32°) of hydroxyapatite were noted (Figure 2E). No pores or obvious hydroxyapatite crystals were observed in the TheraCal LC group on Days 1 and 7 (Figure 2F–I). Diffraction peaks of hydroxyapatite were not observed; instead, diffraction peaks of calcium carbonate (*) and strontium barium zirconate (#) were noted (Figure 2J).

### Degree of conversion

**Figure 3.**
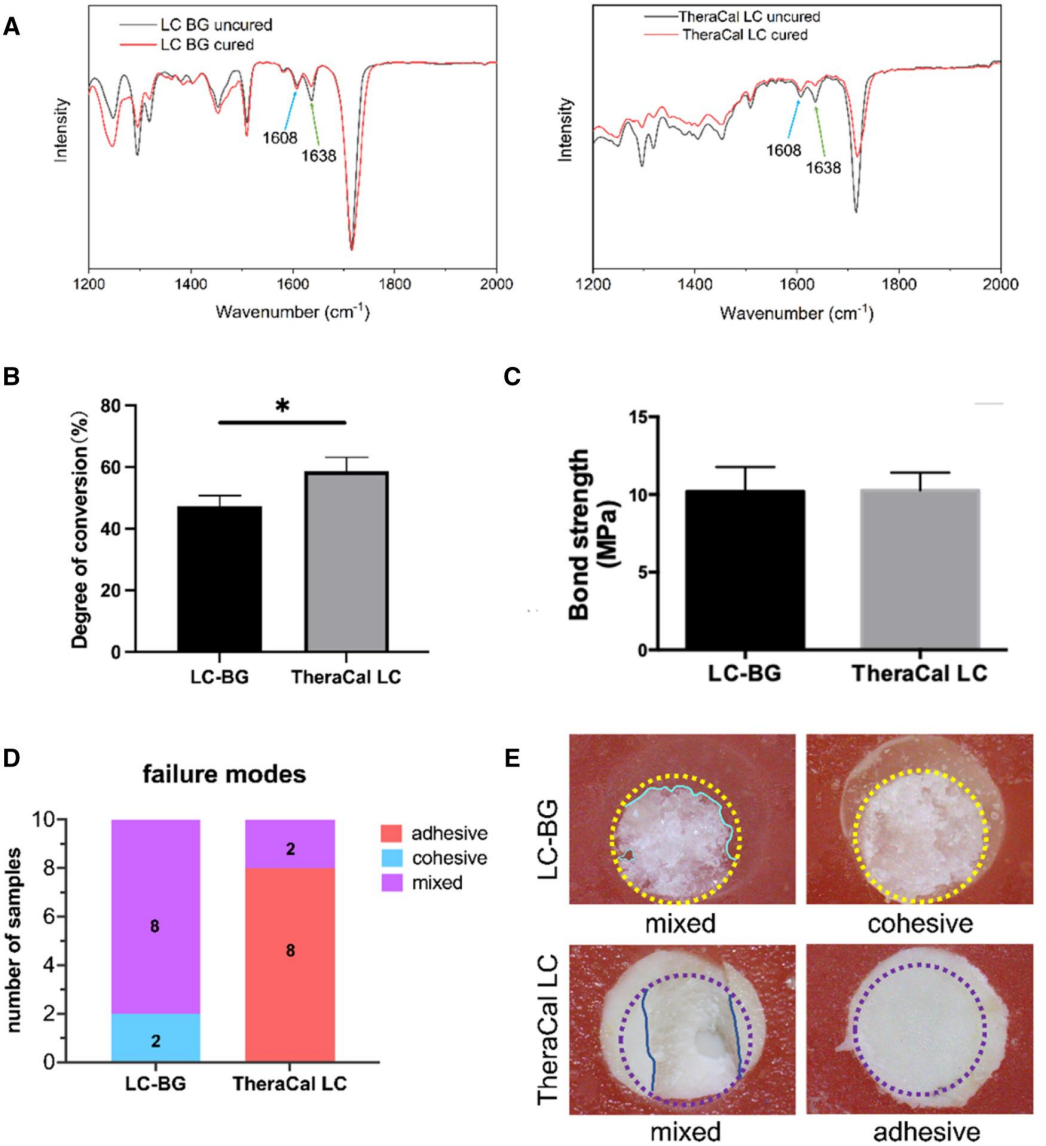
(**A**) ATR-FTIR Spectra of LC-BG and TheraCal LC. A decrease in the absorbance intensities at 1638 cm^−1^ was observed. (**B**) DC of LC-BG and TheraCal LC. (**C**) Shear bond strength between resin and pulp capping materials. (**D**, **E**) Failure modes of LC-BG and TheraCal LC. *P <* *0.05.

The DC of LC-BG was (47.44% ± 3.36%) and DC of TheraCal LC was (58.68% ± 4.60%) (Figure 3A and B). Significant difference was observed between the LC-BG and TheraCal LC groups (*P *=* *0.027).

### SBS of pulp capping materials to resin

As shown in Figure 3C, the SBSs of the LC-BG and TheraCal LC to the resin were (10.19 ± 1.58) MPa and (10.26 ± 1.14) MPa, respectively. No significant difference was observed between the LC-BG and TheraCal LC groups (*P *=* *0.901). As shown in Figure 3D and E, the failure modes of the LC-BG are mixed failure (80%) and cohesive failure (20%), while adhesive failure (80%) was mostly presented in the TheraCal LC group.

### Cytotoxicity of extract on hDPCs

**Figure 4.**
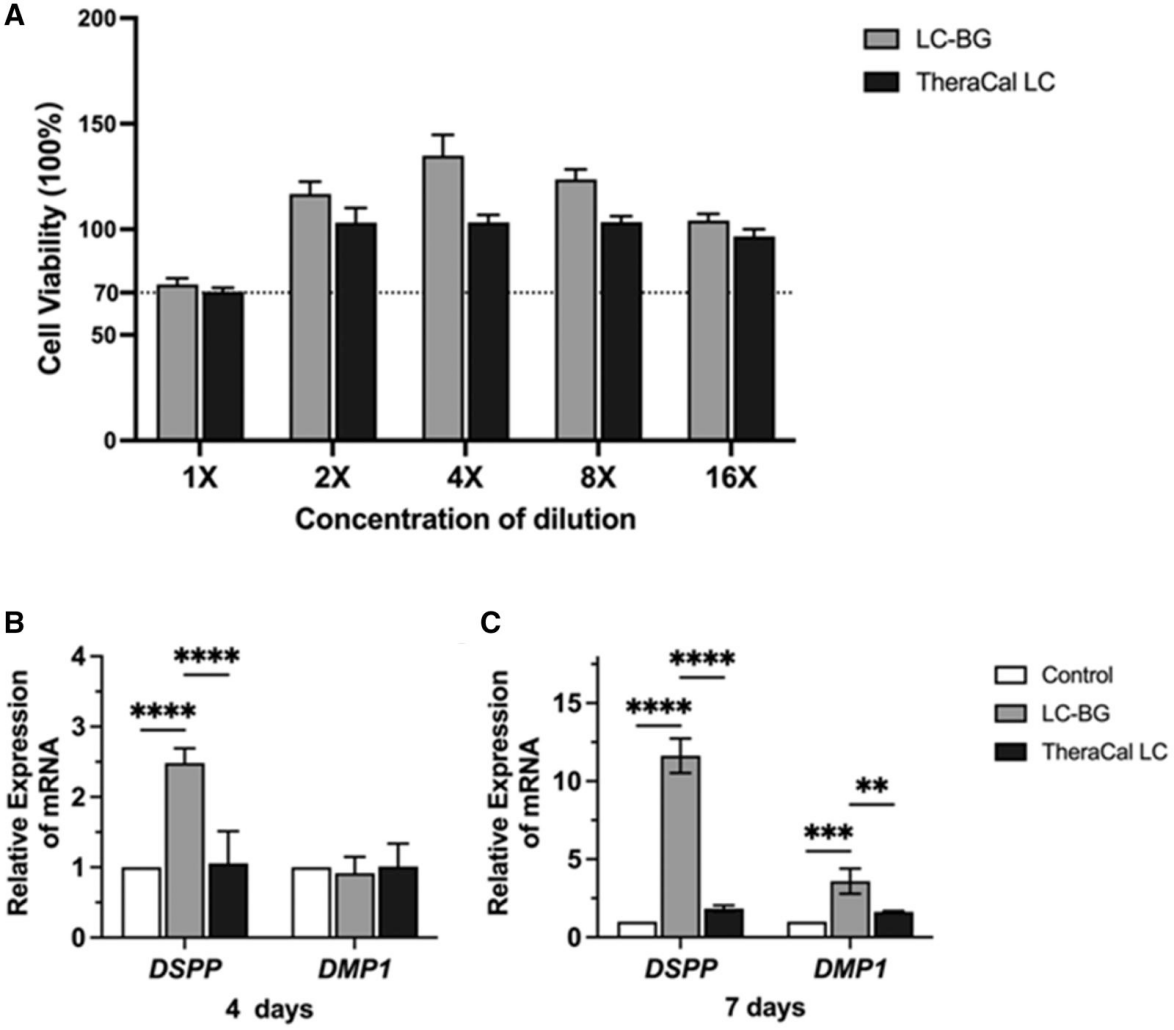
(**A**) CCK-8 Assay revealed that LC-BG and TheraCal LC were not cytotoxic. (**B**, **C**) LC-BG promoted the odontogenic differentiation of hDPCs. The expression of DSPP in hDPCs treated with LC-BG was upregulated at 4 and 7 days. The expression of DMP1 in hDPCs treated with LC-BG was upregulated at 7 days. (****P <* *0.0001, ***P <* *0.001, **P <* *0.01).

The viability of hDPCs treated with LC-BG and TheraCal LC extract is shown in Figure 4A. The viability of the undiluted LC-BG and TheraCal LC groups was more than 70% that of the DMEM group, indicating that LC-BG and TheraCal LC did not have cytotoxic potential according to ISO 10993-5:2009.

### Odontogenic differentiation of hDPCs

As shown in Figure 4B and C, LC-BG promoted the odontogenic-related gene expression of hDPCs: *DSPP* was upregulated in the LC-BG group from Day 4 to Day 7, and the gene expression of *DSPP* in the LC-BG group was higher than in the TheraCal LC and the control groups (*P* < 0.0001). The expression of *DMP1* was upregulated in the LC-BG group on Day 7 and was higher than in the TheraCal LC group (*P *<* *0.01). No significant difference was detected between the TheraCal LC and the control groups.

### Effects on dental pulp tissue after DPC *in vivo*

The numbers of available specimens assessed for the TheraCal LC and LC-BG groups were 9 and 10, respectively, as some of the specimens were damaged during sectioning. Table 2 shows the histological assessment scores of each group. The kappa coefficients between the two observers were 0.90, 0.86, 1, 0.92, and 0.93 for the grading of calcified bridge formation, quality of dentine formation in the bridge, presence of dentine chips, pulpal inflammation and odontoblast-like cell layer formation, respectively.

**Figure 5.**
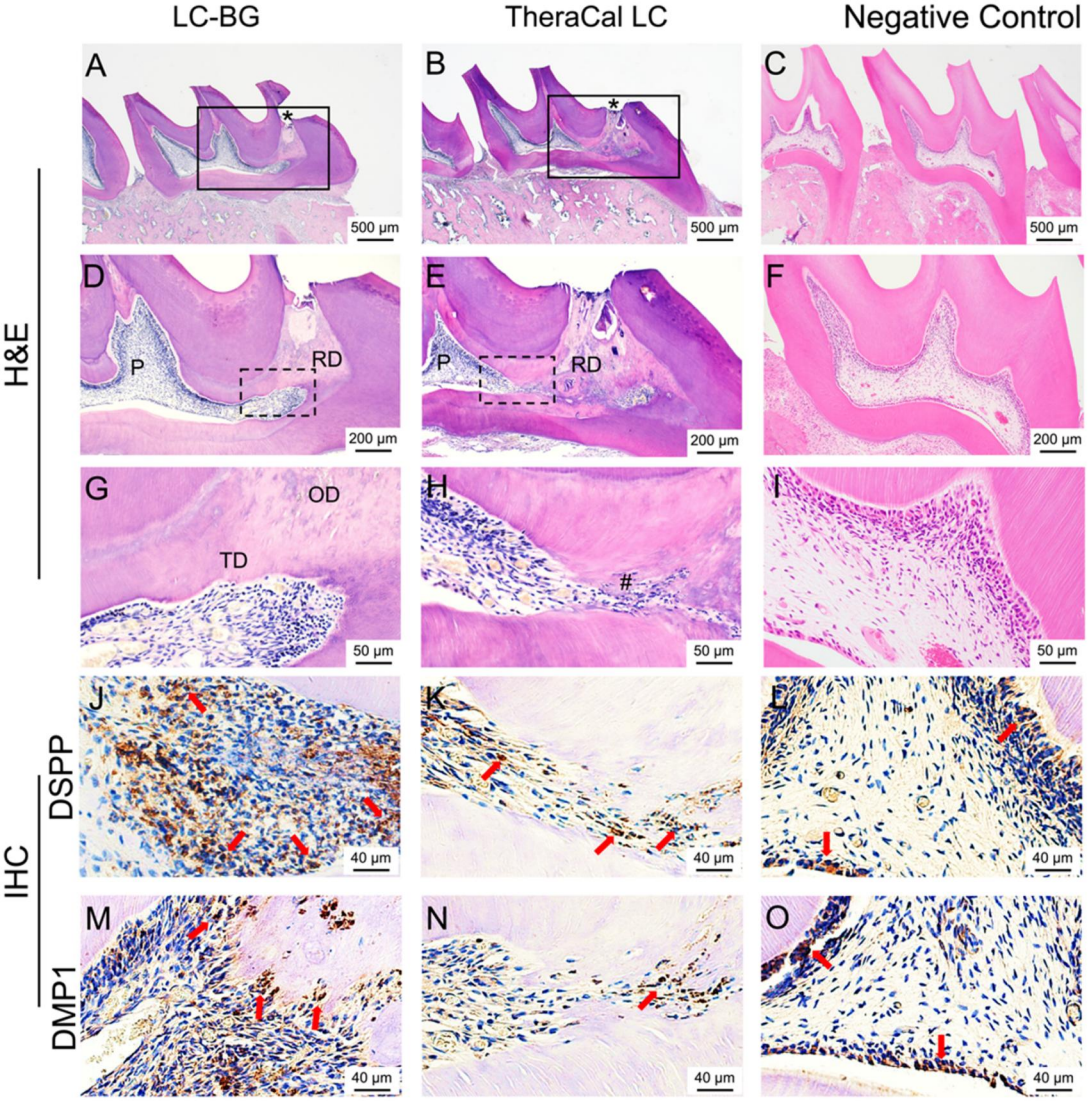
Hematoxylin–eosin staining of the pulp tissues after directly capped with LC-BG (**A**, **D**, **G**), TheraCal LC (**B**, **E**, **H**), and normal pulp (**C**, **F**, **I**) at 4 weeks. D, E, G, H present the rectangular areas in A, B, D, E. Immunohistochemical staining of the pulp tissues after DPC with LC-BG (**J**, **M**), TheraCal LC (**K**, **N**) and normal pulp tissue (**L**, **O**) at 4 weeks. Arrow: DSPP or DMP1-positive cells. P, pulp tissue; *pulp exposure; ^#^inflammatory cell filtration; RD, reparative dentin; OD, osteoid dentin; TD, tubular dentin; OB, odontoblast-like cells.

Eight of the 10 specimens in the LC-BG group exhibited complete calcified bridge formation, mostly with regular or irregular tubular structures (Figure 5A, D and G). However, only three of the nine specimens in the TheraCal LC group formed complete calcified bridge, and two specimens in the TheraCal LC group exhibited disorganized hard tissue formation (Figure 5B, E and H). There were significant differences in calcified bridge formation between the two groups (*P *=* *0.039). Complete odontoblast-like cell layer formation was observed in four specimens in the LC-BG group but in only one specimen in the TheraCal LC group. Compared with the TheraCal LC group, the LC-BG group exhibited better odontoblast-like cell layer formation, but the difference was not significant (*P* = 0.155). Immunohistochemical staining revealed more DSPP-positive and DMP1-positive cells in the LC-BG group than in the TheraCal group (Figure 5J–O).

**Figure 6.**
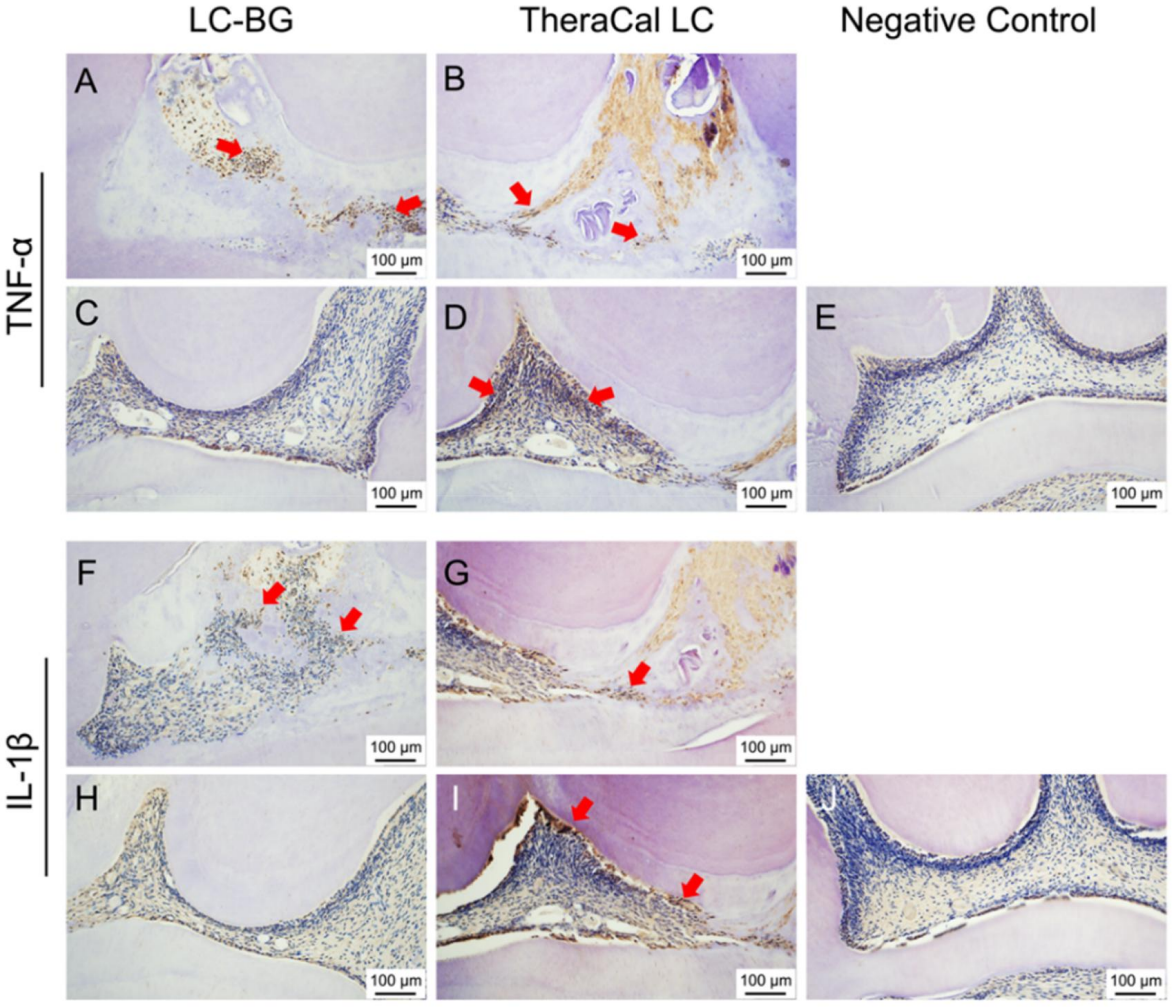
Immunohistochemical staining of the pulp tissues after directly capped with LC-BG (**A**, **C**, **F**, **H**), TheraCal LC (**B**, **D**, **G**, **I**) and normal pulp tissue (**E**, **J**) at 4 weeks. (A, B, F, G) Pulp tissue beneath the mechanical exposure. (C, D, H, I) Pulp tissue distant from pulp exposure. Bar: 100 μm. Arrow: TNF-α- or IL-1β-positive cells.

No to moderate inflammatory responses were detected in the LC-BG group. However, in the TheraCal LC group, 1 specimen exhibited severe inflammation. No significant difference was detected in the inflammatory response between the two groups (*P *=* *0.476). Immunohistochemical staining revealed that IL-1β-positive and TNF-α-positive cells were detected beneath the pulp exposure in both the LC-BG group and the TheraCal LC group (Figure 6A, B, F and G). However, more inflammatory positive cells were present in the TheraCal LC group than in the LC-BG group, and some of the odontoblast cells distant from the pulp exposure presented IL-1β-positive and TNF-α-positive in the TheraCal LC group (Figure 6D and I).

## Discussion

Previously, light-cured calcium silicate was not generally recommended for DPC [28]. Although TheraCal LC is a typical resin-modified calcium silicate, it has good handling characteristics; however, the presence of a resin matrix compromises its bioactivity and biocompatibility. The resin matrix impeded water absorption and hydration of the tricalcium silicate, calcium ion release and calcium hydroxide formation were hindered, and biomineralization was compromised [13–16]. TheraCal LC exhibited poor results on pulpotomy and partial pulpotomy [29–31]. Moreover, TheraCal LC induced slower and thinner dentine bridge formation than Biodentine and ProRoot^®^ MTA (WMTA; Dentsply, USA) did after DPC [19, 32]. After DPC on human permanent teeth with deep caries, the success rate of TheraCal LC was similar to Dycal and lower than BioMTA+ (Cerkamed, Poland) and Biodentine [11, 12].

To improve the bioactivity of the light-cured material, we constructed a hygroscopic composite by encapsulating BG into PEG. The ether oxygen atom (–O–) and the terminal hydroxyl group (–OH) on the main chain of oxyethylene polymerization can react with water to form hydrogen bonds, so PEG can absorb water even when mixed inside the hydrophobic agent [23]. Our study proved that, after soaking in SBF, the PEG microparticles were instantly dissolved, interconnecting pores formed, and HA crystals coating on the BG microparticles were detected by SEM and XRD. However, TheraCal LC presented no pores or HA formation. These results also corresponded to ion release. LC-BG exhibited faster ion release and more ion exchange. More Ca and Si release was detected in the LC-BG group, and a large amount of ${\text{PO}}_{4}^{3-}$ from SBF was consumed to form HA crystals. We proved that PEG could absorb SBF into the composite, benefiting the ion release and hydration process of BG.

To precisely control pulp damage and weaken the effect of uncured monomers, we chose a DPC model in rats to investigate pulp responses. Dammaschke [33] reported that rat molar teeth exhibited similar pulp tissue biological reactions and wound healing procedures after DPC with other mammals, but the size of the teeth may bring some difficulties for surgery. To overcome these difficulties, our surgery was performed under a dental microscope to precisely control the size of the pulp exposure. In this study, the histologic analysis revealed that a complete dentin bridge was induced in only three specimens in the TheraCal LC group; two specimens exhibited disorganized hard tissue formation, and one specimen exhibited severe inflammatory responses. The results were not as good as those of previous studies [27, 34], but were in accordance with those of Park’s study [32]. The use of different animal models and sample sizes may explain these differences. Our results indicated that the pulp response to TheraCal LC was not steady, which may affect the long-term success of the therapy.

The traditional melt-derived BG displayed low porosity, small surface area and smooth texture and exhibited unsatisfactory pulp responses after pulp capping [35, 36]. With technological advancements, sol-gel-derived BG has shown better bioactivity and better results after DPC. No or slight inflammatory pulp responses and complete, organized dentin bridge formation were observed. The BG used in our study is a kind of sol-gel-derived BG synthesized with a phosphorus precursor named phytic acid. It was selected as the bioactive component because our previous study demonstrated that it was effective in pulp repair and regeneration [20, 21]. For the LC-BG group, 8 of the 10 specimens induced tubular and complete dentin bridge, either regular or irregular. Polarizing odontoblast-like cells were also detected lining beneath the tubular dentin bridge. The dentin bridge beneath the pulp exposure exhibited low mineralization with some defects and cell inclusions, indicating that the reparative process induced by LC-BG was relatively rapid. Moreover, the layer near the pulp was well-organized and highly mineralized. In one specimen, a layer of osteoid dentin with a lacunar structure was observed between these two layers (Figure 5G). Compared with TheraCal LC, LC-BG had significantly better results in calcified bridge formation, and the differences were significant, indicating that LC-BG induced better biomineralization. Immunohistochemical staining also proved better odontogenic protein (DSPP and DMP1) expression in the LC-BG group and was in accordance with odontogenic gene expression *in vitro*.

Some of the LC-BG and TheraCal LC specimens presented mild to severe pulpal inflammation, and immunohistochemical staining was carried out for further investigation of inflammation. Notably, IL-1β-positive and TNF-α-positive cells were detected only beneath the pulp exposure in the LC-BG group (Figure 5B and G) but were detected in some of the odontoblast cells distant from the exposure site in the TheraCal LC group (Figure 5D and I). This result may indicate that the TheraCal LC induced more extensive pulpal inflammation than did the LC-BG. One limitation of this animal study is that the teeth were sound and the pulp was not infected. Future experiments could simulate caries-related exposure. On the other hand, we found that pulp inflammation is still a problem, and the DC of LC-BG and TheraCal indicated residual monomers after light curing. The next step is focused on reducing inflammation, which might be caused by residual monomers [18, 27, 37–41].

Resin composites are recommended for the restoration of the cavity after DPC. The interface between restorative resin and the pulp capping material is important for the longevity and predictability of the restoration [42], so the right time for restoration is after the initial setting of the pulp capping materials. The prolonged setting time and poor bond strength to resin are the major drawbacks of bioceramics. Studies have shown that the bond strength between resin and MTA was lower than the resin-based TheraCal LC [7, 43]. Our study indicated that after initial setting, the SBSs of the LC-BG and TheraCal LC groups were not significantly different. Therefore, LC-BG has good instant curing and bonding properties because of its resin-based nature, which can avoid second visits and may improve the longevity and predictability of subsequent restoration. The most common type of failure in the LC-BG was mixed failure, whereas that in the TheraCal LC was adhesive failure. These findings indicate that the cohesive strength of TheraCal LC was better than that of LC-BG.

In conclusion, within the limitations of our study, the newly developed hygroscopic LC-BG composite showed better bioactivity and odontogenic differentiation than TheraCal LC did *in vitro* and induced better integrity of calcified bridge than the TheraCal LC did *in vivo* for the 4-week observational period. The null hypothesis is rejected. This composite has a potential to be an effective DPC material. Future studies could investigate pulp responses in a caries-related model for longer observation periods.

## Supplementary Material

rbae114_Supplementary_Data
