# Supplementary figures and images for: Hygroscopic bioactive light-cured composite promoting dentine bridge formation
Source: Regen Biomater. 2024 Sep 26;11:rbae114. doi: 10.1093/rb/rbae114 (PMC11467188; doi:10.1093/rb/rbae114)

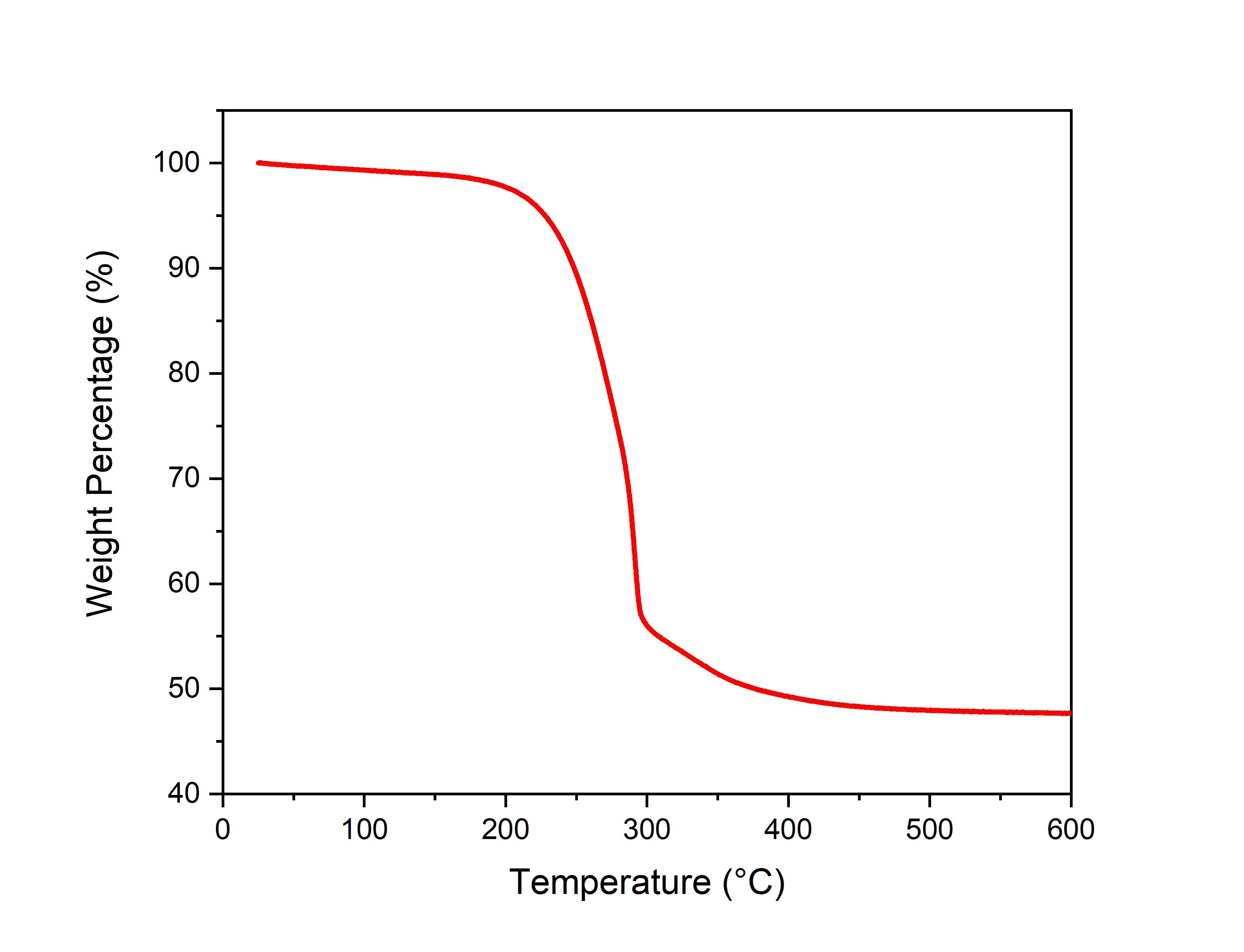

Supplement: rbae114_Supplementary_Data [file rbae114_supplementary_data.jpeg]
